# Refining outcome prediction after traumatic brain injury with machine learning algorithms

**DOI:** 10.1038/s41598-024-58527-4

**Published:** 2024-04-05

**Authors:** D. Bark, M. Boman, B. Depreitere, D. W. Wright, A. Lewén, P. Enblad, A. Hånell, E. Rostami

**Affiliations:** 1 Department of Medical Sciences Neurosurgery, Uppsala University, Uppsala, Sweden; 2 Division of Clinical Epidemiology, Department of Medicine Solna, Stockholm, Sweden; 3 Department of Clinical Epidemiology, Karolinska Institutet, Stockholm, Sweden; 4 Department of Neurosurgery, University Hospitals Leuven, Leuven, Belgium; 5 Department of Emergency Medicine, Emory University, Atlanta, Georgia; 6 Department of Neuroscience, Karolinska Institutet, Stockholm, Sweden

**Keywords:** Computational biology and bioinformatics, Neuroscience, Medical research, Neurology

## Abstract

Outcome after traumatic brain injury (TBI) is typically assessed using the Glasgow outcome scale extended (GOSE) with levels from 1 (death) to 8 (upper good recovery). Outcome prediction has classically been dichotomized into either dead/alive or favorable/unfavorable outcome. Binary outcome prediction models limit the possibility of detecting subtle yet significant improvements. We set out to explore different machine learning methods with the purpose of mapping their predictions to the full 8 grade scale GOSE following TBI. The models were set up using the variables: age, GCS-motor score, pupillary reaction, and Marshall CT score. For model setup and internal validation, a total of 866 patients could be included. For external validation, a cohort of 369 patients were included from Leuven, Belgium, and a cohort of 573 patients from the US multi-center ProTECT III study. Our findings indicate that proportional odds logistic regression (POLR), random forest regression, and a neural network model achieved accuracy values of 0.3–0.35 when applied to internal data, compared to the random baseline which is 0.125 for eight categories. The models demonstrated satisfactory performance during external validation in the data from Leuven, however, their performance were not satisfactory when applied to the ProTECT III dataset.

## Introduction

TBI remains one of the leading causes of morbidity and mortality worldwide^1^ and those who survive present with a variety of symptoms and reduction in function that are difficult to predict. Clinicians treating patients often make therapeutic decisions based on their assessment of prognosis. According to a 2005 survey, 80% of doctors believed that an accurate assessment of prognosis was important when they made treatment decisions for TBI patients, yet only a third of doctors thought that they could accurately assess the prognosis^2^. Multiple prognostic models for TBI have accumulated over the last decades but none of them are widely used in clinical practice. In a systematic review by Perel et al. 2005, a total of 53 reports were identified including 102 models^3^. They conclude that while there are many prognostic models, they typically have several limitations such as small sample size, that they are mainly developed in high income countries, that they have no external validation, and that they have limited clinical user friendliness. A major leap forward was made between 2007 and 2008 when the Corticosteroid Randomisation After Significant Head Injury (CRASH) collaborators as well as International Mission for Prognosis and Clinical Trial (IMPACT) developed prognostic models and used each other’s data for external validation. The IMPACT study included 8686 TBI patients and the CRASH study had 10,008 patients with the outcome measure assessed as mortality at 14 days and death or severe disability at 6 months respectively^4,5^. Although the discriminative abilities of both models are quite good, with an area under the receiver operator curve (AUROC) of around 0.8, the outcome prediction is dichotomized into poor versus good outcome. This not only hampers individual health care planning but also limits the level of detail that can be provided to patients and/or their caregivers. Furthermore, a dichotomized outcome prediction tool limits the ability to stratify patients for clinical studies as well as for precise evaluation of the effect of clinical and pharmacological interventions. A prediction model that provides the full 1–8 scale of Glasgow outcome scale extended (GOSE) could improve the fidelity of the tool.

In recent years, advanced machine learning algorithms are increasingly utilized in data analysis throughout medical research. Eloranta et al. have provided a review of machine learning for clinicians without a technical computer science background^6^. Furthermore, complex machine learning models such as neural networks have recently received increased attention, for example in image recognition and segmentation, which is particularly useful in radiology^7^. Classically dichotomized prediction models typically use logistic regression and are evaluated with AUROC. The goal of this study was to explore other advanced methods for training and evaluating multi-class machine learning models in order to develop a well calibrated and clinically user-friendly prediction model for TBI that allows for full 8-category GOSE prediction.

## Results

**Figure 1:**
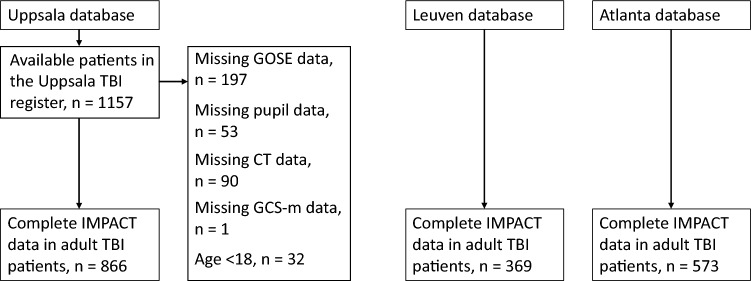
Flowchart of patient inclusion, including exclusion criteria of the Uppsala cohort.

A total of 1157 patients were considered for inclusion from the Uppsala TBI registry^8^. The patients had a mean admission GCS of 9.6 (SD 3.4). In the Uppsala cohort, a total of 324 patients (37%) had severe TBI (GCS 3–8), 398 (46%) had moderate TBI (GCS 9–13), and 144 (17%) had mild TBI (GCS 14–15). All included patients were suffering from acute TBI, however in some patients there was a slight delay when patients were initially treated in the region hospital. The mean time from trauma to GCS evaluation was 1.37 days (SD 12.8) in the Uppsala cohort. No data was imputed, and the data was restricted to complete cases with all input variables which resulted in the inclusion of 866 patients. There were some GOSE class imbalances. In the training data 122 patients (26%) had an outcome of GOSE 8, 78 patients (17%) GOSE 7, and 77 patients (16%) GOSE 3. External validation was performed in 369 TBI patients with complete datasets from Leuven (Belgium) and 573 TBI-patients extracted from ProTECT III trial (US) (Fig. 1)^18^. Patient demographics are presented in Table 1. The median age of the Uppsala cohort was 53 (IQR = 31). The median age of the Leuven cohort was significantly higher at 63 (IQR = 35, p < 0.001) while the median age of the ProTECT cohort was significantly lower at 35 (IQR = 28, p < 0.001).

**Table 1:** Patient demographics of the included patients from the Uppsala traumatic brain injury register.

Trauma mechanism	n	Admission data	n	Radiology findings	n	Marshall score	n	GOSE	N
Fall accident	446 (52%)	Male	668 (77%)	ASDH	273 (32%)	Diffuse I	12 (1%)	1	126 (15%)
Vehicle accident	214 (25%)	Female	198 (23%)	Contusion	222 (26%)	Diffuse II	392 (45%)	2	10 (1%)
Pedestrian hit by vehicle	30 (3%)	Mean age	52.6	Traumatic SAH	72 (8%)	Diffuse III	113 (13%)	3	158 (18%)
Cyclist hit by vehicle	28 (3%)	Median age	56	EDH	67 (8%)	Diffuse IV	51 (6%)	4	65 (8%)
Assault	38 (4%)	GCS-m 1	32 (4%)	DAI	38 (4%)	Evacuated mass lesion	177 (20%)	5	88 (10%)
Sports accident	24 (3%)	GCS-m 2	27 (3%)	Impression fracture	21 (2%)	Non-evacuated mass lesion	121 (14%)	6	87 (10%)
Other	86 (10%)	GCS-m 3	268 (31%)	Mixed	141 (16%)			7	131 (15%)
		GCS-m 4	38 (4%)	Other	23 (3%)			8	201 (23%)
		GCS-m 5	103 (12%)	Normal	5 (1%)				
		GCS-m 6	398 (46%)						
		Both pupils reacting	748 (86%)						
		One pupil reacting	61 (7%)						
		No pupil reacting	57 (7%)						

*GCS-m* Glasgow Coma Scale-motor, *ASDH* acute subdural hematoma, *SAH* subarachnoid hemorrhage, *EDH* epidural hematoma, *DAI* diffuse axonal injury.

### Model performance

The discrimination and calibration reported below are the results of the performance in the test set and external validation sets. The complete results from the tenfold cross validation can be found in supplementary Table 1. A summary of the performance of each model is presented in Table 2.

**Table 2:** Summary of the models’ performances in the internal dataset.

	Accuracy	AW1	AW2	ABA	Precision	Recall	Specificity	MD	SDD	TCPD	MCPD	AUROC-U	AUROC-M
Uppsala													
POLR	0.30	0.45	0.66	0.54	0.35	0.30	0.82	0.35	0.29	0.98	0.28	0.77	0.80
RF	0.35	0.49	0.69	0.56	0.32	0.35	0.83	0.89	0.32	0.88	0.39	0.77	0.83
NN	0.35	0.50	0.64	0.57	0.31	0.35	0.85	0.23	0.38	0.64	0.23	0.75	0.83
Leuven													
POLR	0.44	0.59	0.74	0.57	0.65	0.44	0.83	−0.54	0.19	0.78	0.33	0.90	0.88
RF	0.51	0.67	0.79	0.56	0.43	0.51	0.73	0.64	−0.04	0.81	0.28	0.89	0.86
NN	0.52	0.69	0.78	0.56	0.50	0.52	0.75	0.53	−0.01	0.74	0.25	0.86	0.78
ProTECT III													
POLR	0.18	0.23	0.41	0.51	0.16	0.18	0.84	−2.53	−0.58	1.20	0.38	0.59	0.63
RF	0.20	0.26	0.45	0.51	0.30	0.20	0.85	−2.15	−0.80	1.21	0.45	0.51	0.60
NN	0.21	0.29	0.44	0.52	0.12	0.21	0.86	−2.35	−1.02	1.14	0.34	0.59	0.69

*LR* linear regression, *POLR* proportional odds logistic regression, *RF* random forest regression, *NN* neural network, *AW1* accuracy within one category, *AW2* accuracy within two categories, *ABA* average balanced accuracy, *MD* mean discrepancy, *SSD* standard deviation discrepancy, *MMP* mean maximum probability, *TCPD* total category proportion discrepancy, *MCPD* maximum category proportion discrepancy, *AUROC-M* area under the receiver operator curve for mortality (GOSE 1), *AUROC-U *area under the receiver operator curve for unfavorable outcome (GOSE 1–4).

In our analysis, we compared the performance of three models: Proportional odds logistic regression (POLR), Random Forest (RF) and Neural Network (NN), across different metrics and cohorts.

Accuracy was relatively similar in all three models, with POLR slightly underperforming compared to RF and NN. Specifically, POLR performed with an accuracy of 0.3, 0.44 and 0.18 in the Uppsala, Leuven, and ProTECT III cohorts, respectively. While RF and NN performed with an accuracy of 0.35, 0.51–0.52, and 0.20–0.21 in the Uppsala, Leuven and ProTECT III cohorts. Both accuracy within one (AW1) and two categories (AW2) follow a similar pattern, with RF and NN outperforming POLR.

All models performed better in the Leuven cohort, and worse in the ProTECT III cohort. Interestingly, the Average Balanced Accuracies (ABA) were quite similar between all models and cohorts, around the suboptimal values of 0.5–0.6. Contrarily, POLR exhibited slightly better precision in the Uppsala and Leuven cohorts, while the precision of the RF model was best in the ProTECT III cohort, meaning that they had less risk of false positives in those cohorts. The recall rates were comparable in all models, with values ranging from 0.3 to 0.35, 0.44–0.52, and 0.18–0.21 in the Uppsala, Leuven and ProTECT III cohorts, respectively, indicating similar tendencies to make false negative predictions.

Specificity was roughly equal when evaluated in the Uppsala (0.82–0.85) and the ProTECT III (0.84–0.86) cohorts. In the Leuven cohort, POLR achieved higher specificity (0.83) compared to RF (0.73) and NN (0.75).

NN showed the best mean deviation (MD) at 0.23 in the Uppsala cohort, suggesting balanced predictions, followed by POLR (0.35) and RF (0.89), with the latter being slightly more optimistic. In external validation, NN and RF performed similarly in the Leuven cohort, with MD values of 0.53 and 0.64, respectively. POLR, however, had a pessimistic tendency (−0.54). All models performed overly pessimistic in the ProTECT III cohort with MDs ranging between −2.15 to −2.53.

The SDD values indicate that predictions in the Uppsala and Leuven cohorts tended towards the extremes of the GOSE scale. Conversely, in the ProTECT III cohort, NN performed more predictions in the center of the scale (SDD −1.02) compared to RF (−0.8) and POLR (−0.58).

TCPD revealed that all models have similar risk of overpredicting some categories, but that NN was slightly more balanced compared to POLR and RF in all cohorts. MCPD, however, indicated that all models were equally likely to over- or underpredict a specific category.

Finally, the Area Under the Receiver Operating Characteristic (AUROC) for unfavorable outcome and mortality were similar across all models. AUROC values for unfavorable outcome ranged from 0.75–0.77, 0.86–0.9, and 0.51–0.59 in the Uppsala, Leuven, and ProTECT III cohorts respectively. For mortality the values ranged from 0.8–0.83, 0.78–0.88, and 0.60–0.69 in the Uppsala, Leuven, and ProTECT III cohorts respectively.

**Figure 2:**
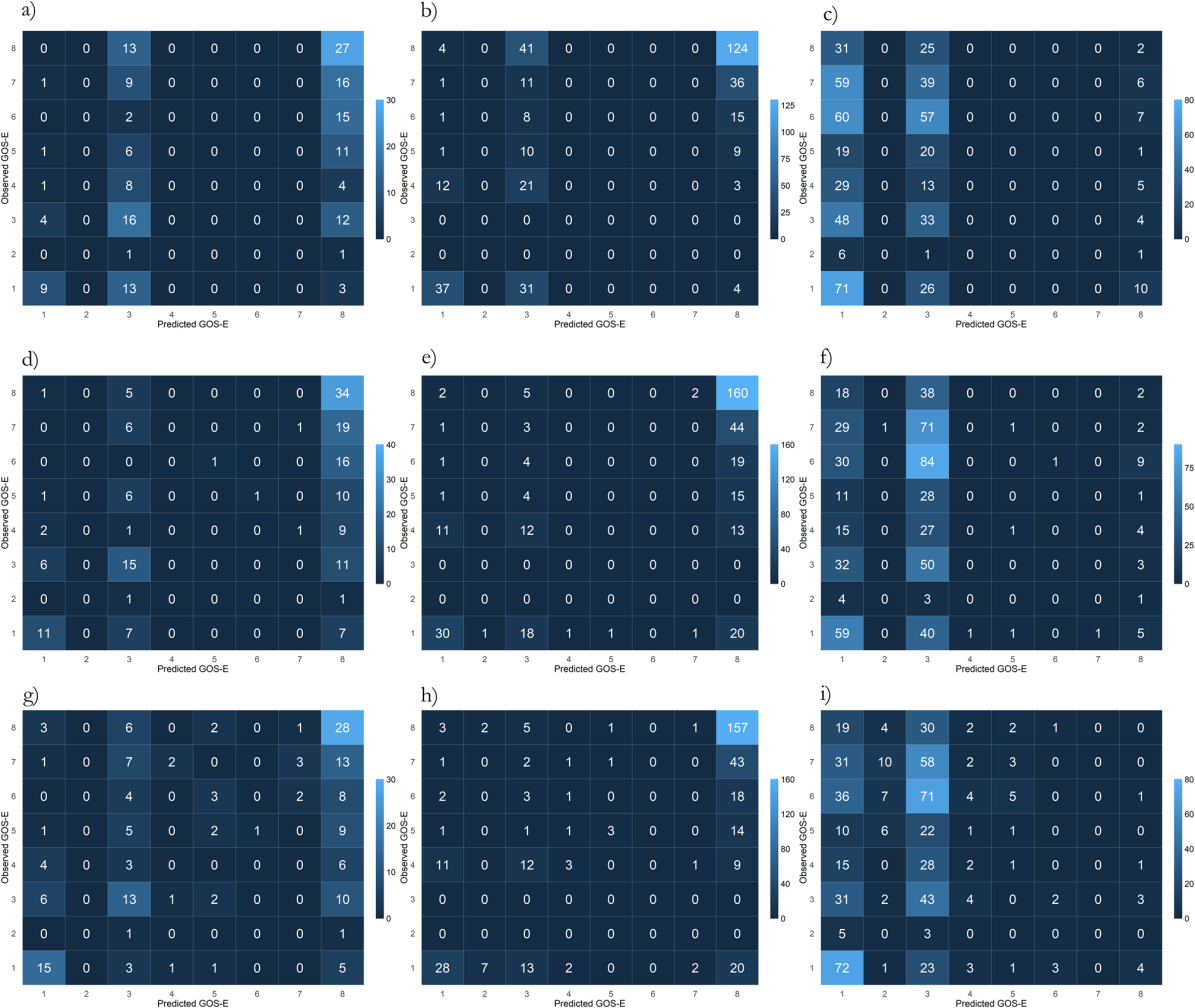
Confusion matrix evaluation of the models, showing observed Glasgow outcome scale extended (GOSE) over predicted GOSE. All models overpredict GOSE categories 1, 3 and 8. While the proportional odds logistic regression (POLR) model only predicts those two categories, the random forest (RF) and neural network (NN) models predict some middle categories. (**a**) POLR in the Uppsala cohort showing, albeit somewhat balanced, prediction of only GOSE 1, 3, and 8. (**b**) POLR in the Leuven cohort showing overprediction of GOSE 8. (**c**) POLR in the ProTECT III cohort showing overprediction of GOSE 1 and 3. (**d**) RF in the Uppsala cohort showing overprediction of GOSE 8, some in GOSE 1 and 3, and a few middle category predictions. (**e**) RF in the Leuven cohort, similarly overprediction GOSE 8. (**f**) RF in the ProTECT III cohort, overpredicting GOSE 1 and 3. Finally, (**g**) shows the NN model with slightly more spread-out predictions but still overprediction of GOSE 1, 3 and 8. (**h**) The NN model overpredicts GOSE 8 in the Leuven cohort. (**i**) The NN model also overpredicts GOSE 1 and 3 in the ProTECT III cohort.

**Figure 3:**
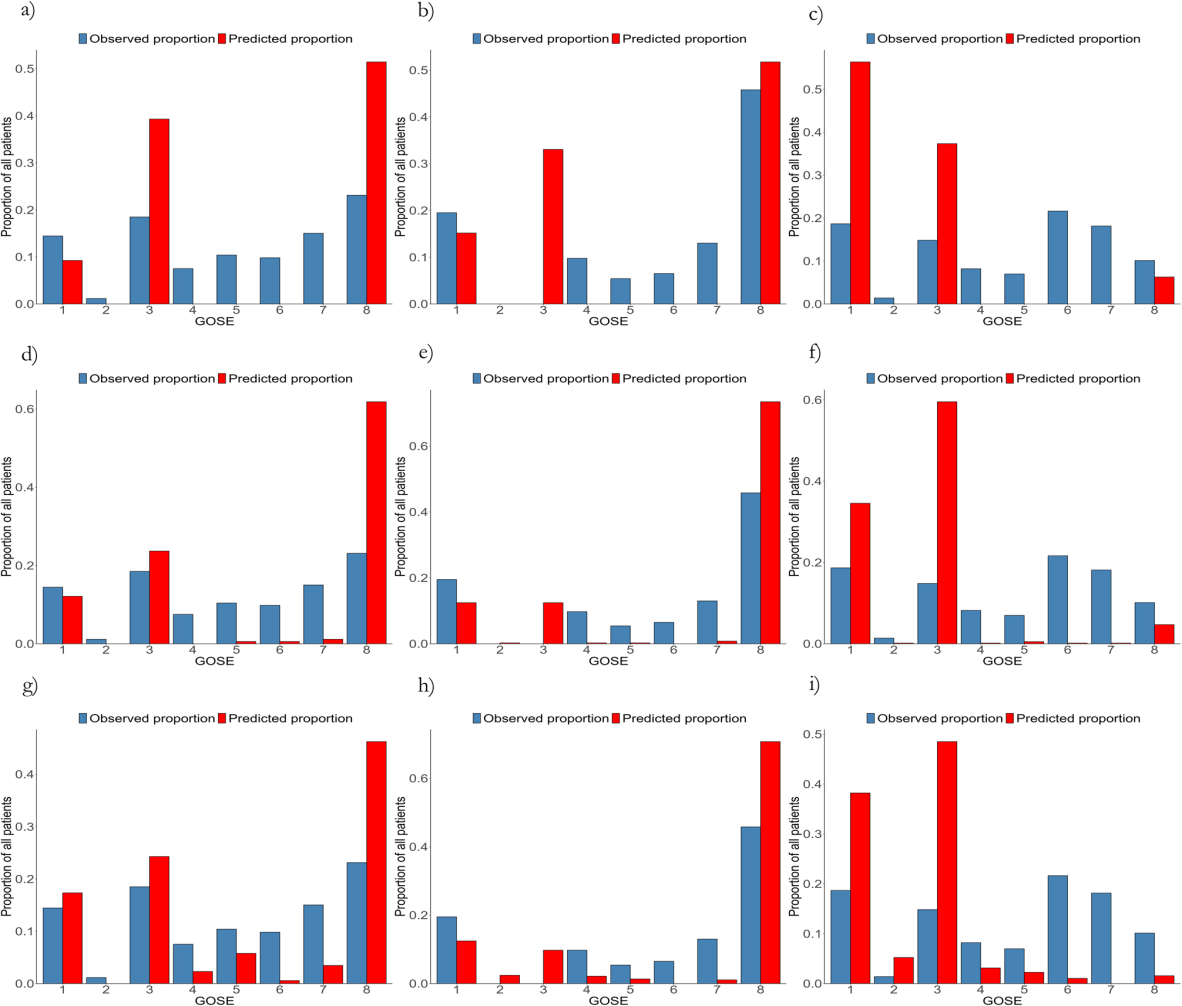
Category proportion graphs of the proportional odds logistic regression (POLR), random forest (RF) and neural network (NN) models complementing the confusion matrices showing clearly the overprediction of Glasgow outcome scale extended (GOSE) categories 1 3, and 8 in all models with the neural network (**g–i**) showing slightly more spread-out predictions. The rows correspond to the POLR (**a–c**), RF (**d–f**) and NN (**g–i**) models and the columns to the Uppsala (**a,d,g**), Leuven (**b,e,h**) and ProTECT III (**c,f,i**) cohorts respectively.

**Figure 4:**
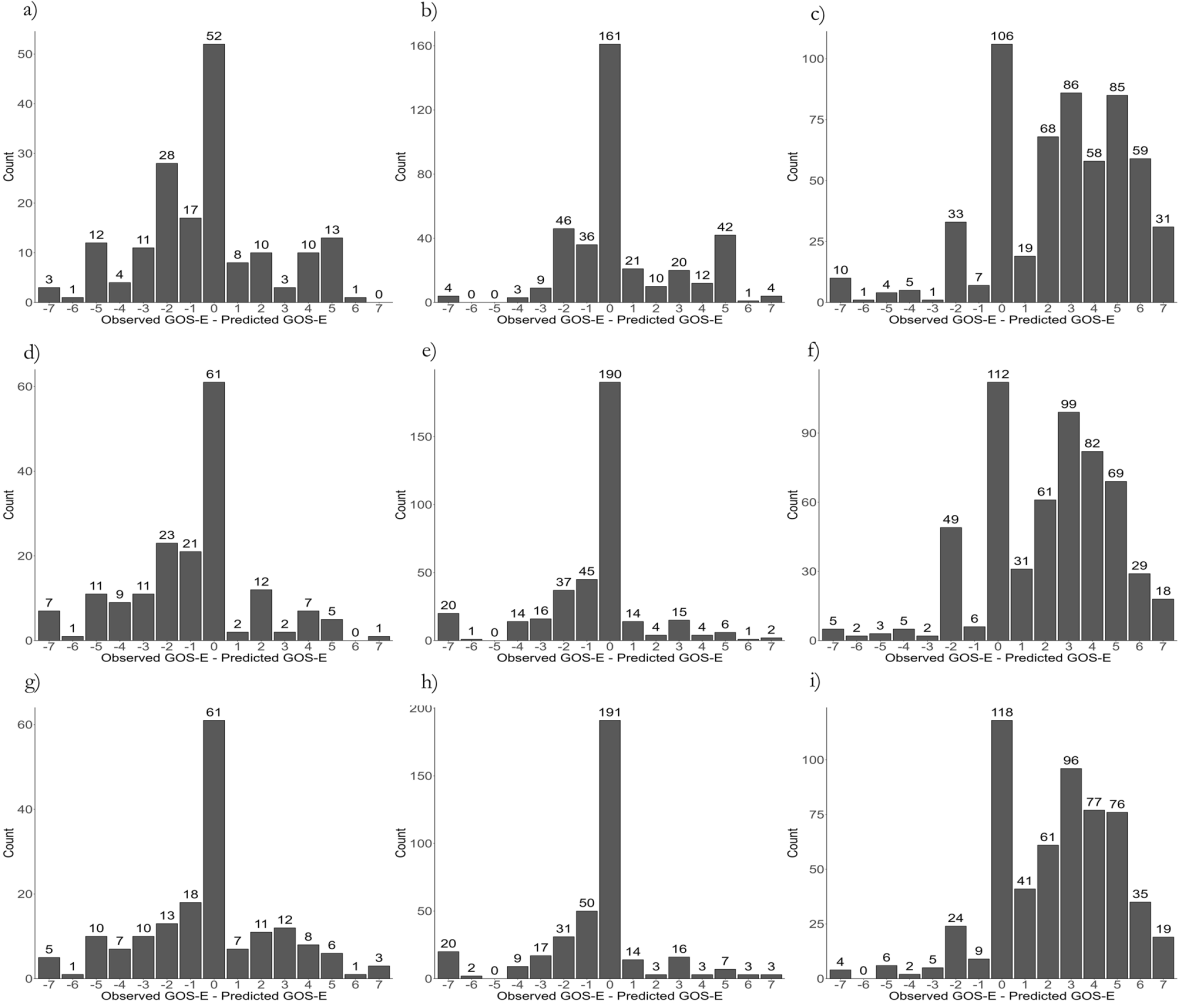
Error distribution graphs for the models’ proportional odds logistic regression (POLR) in (**a–c**), random forest (RF) in (**d–f**), and neural network (NN) in (**g–i**). The graphs visualize the observed Glasgow outcome scale extended (GOSE) minus the predicted GOSE, and thus the balance of the models. All models slightly overestimated outcome in the Uppsala cohort (**a,d,g**), were fairly balanced in the Leuven cohort (**b,e,h**) and underestimated outcome in the ProTECT III cohort (**c,f,i**).

Graphically, the results are displayed as confusion matrices in Fig. 2, as category proportion graphs in Fig. 3 and as error distribution graphs in Fig. 4. The confusion matrices and category proportion graphs clearly show that POLR only predicted GOSE 1, 3, and 8 with an overprediction of GOSE 8 in the Uppsala and Leuven cohorts and overprediction of GOSE 1 in the ProTECT III cohort. RF and NN similarly overpredicted GOSE 1, 3, and 8 but made some predictions in the other categories as well. They also tend to overpredict category 8 in the Uppsala and Leuven cohorts, and GOSE 3 in the ProTECT III cohort. The error distribution graph shows that the models, however, were fairly balanced overall in the Uppsala and Leuven cohorts but that they were too pessimistic in the ProTECT III cohort.

**Figure 5:**
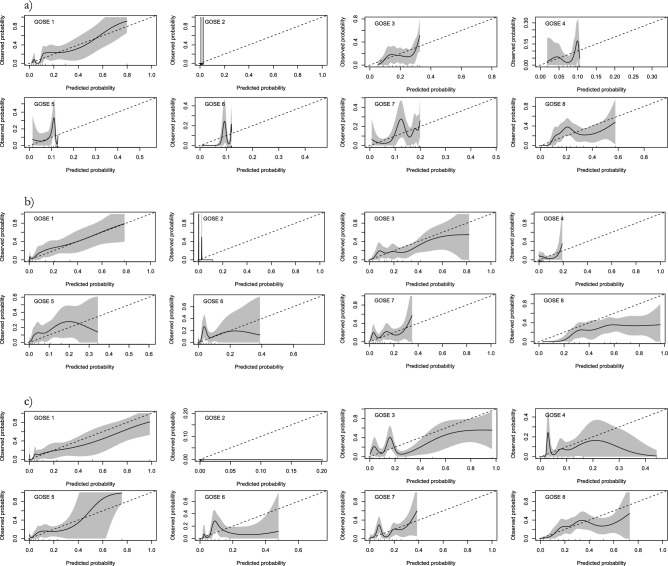
Calibration graphs of the proportional odds logistic regression (POLR), random forest (RF) and neural network (NN) models in the Uppsala cohort. The solid line describes the performance of the models in relation to the observed probability for that category, with the grey area representing two standard errors. The dashed line represents an optimally calibrated model, where the area over the thick line represents underestimation, and the area under the thick line represents overestimation of the probability for a patient to end up in the respective category. Note that the y-axis differs in between some of the Glasgow outcome scale extended (GOSE) categories. (**a**) The POLR models predicted probability is fairly calibrated in GOSE 1 and 8 categories but cuts short at around 0.1–0.2 in category 3–7 and practically never predicts GOSE 2. (**b**) The RF model’s predicted probability is fairly calibrated in GOSE categories 1 and 3 while overpredicting GOSE 8 and under predicting GOSE 4–7 and giving a very low predicted probability for GOSE 2. (**c**) Similarly, the NN model is fairly calibrated in GOSE 1, 3, and 8 while adding better predicted probability for GOSE 4 and 5. The NN still underpredicts GOSE 2, 6, and 7.

The POLR, RF, and NN models were assessed in the Uppsala cohort using calibration plots (Fig. 5). The calibration plots revealed that the POLR model demonstrated reasonable calibration in the GOSE categories 1 and 8. However, it underpredicted the probabilities for categories 3–7, and all models practically predicted no probability for GOSE 2. The RF models exhibited sufficient calibration in the GOSE categories 1 and 3, accurately predicting the probabilities. However, they tended to overpredict GOSE 8 and underpredict GOSE 4–7. Furthermore, the models showed very low predicted probabilities for GOSE 2. Likewise, the NN model is reasonably calibrated in the GOSE categories 1, 3, 8 while adding better calibration in predicted probability for GOSE 4 and 5 when compared to RF and POLR. However, the NN still underpredicted the GOSE categories 2, 6, and 7.

**Figure 6:**
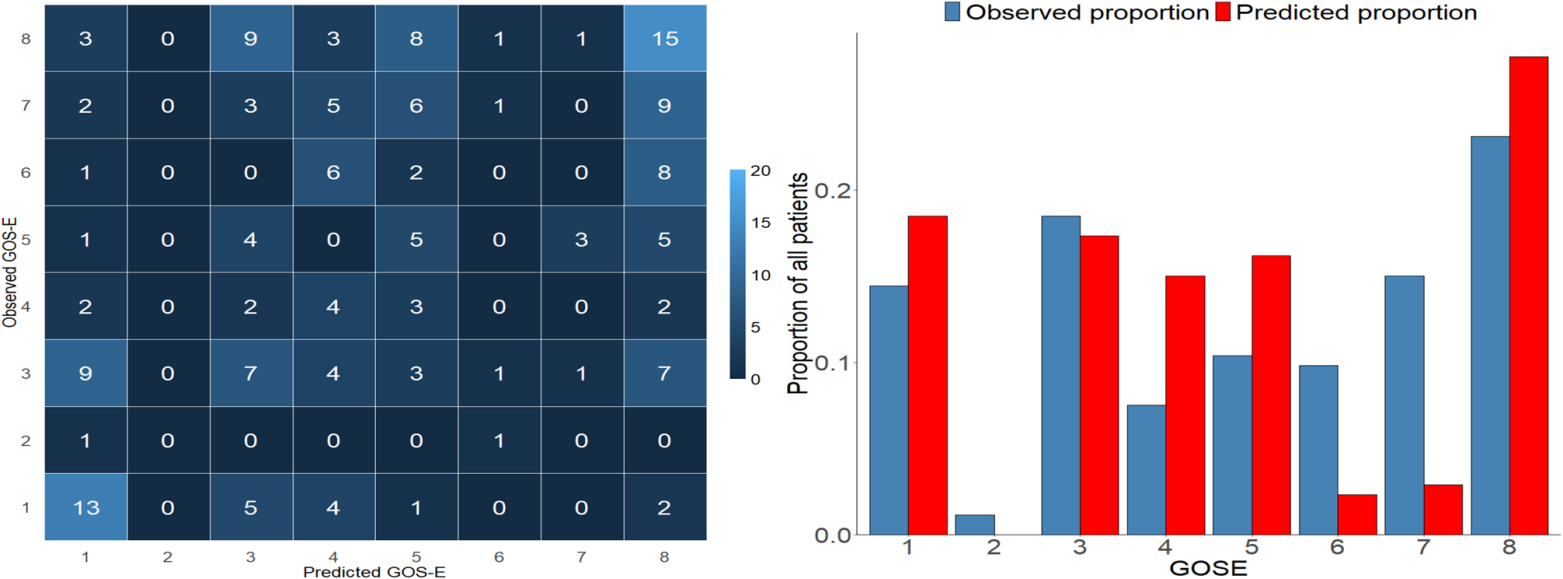
An ensemble of 10,000 proportional odds logistic regression (POLR) models trained on data that were trained using a balanced subsample of the data. The graphs display more stratified prediction of all categories of the Glasgow outcome scale extended except category two.

Balanced undersampling improved the stratification of the models, and an ensemble of 10,000 POLR models trained on a more balanced dataset made predictions in all GOSE categories except GOSE 2 (Fig. 6). However, accuracy slightly decreased to 0.28.

**Figure 7:**
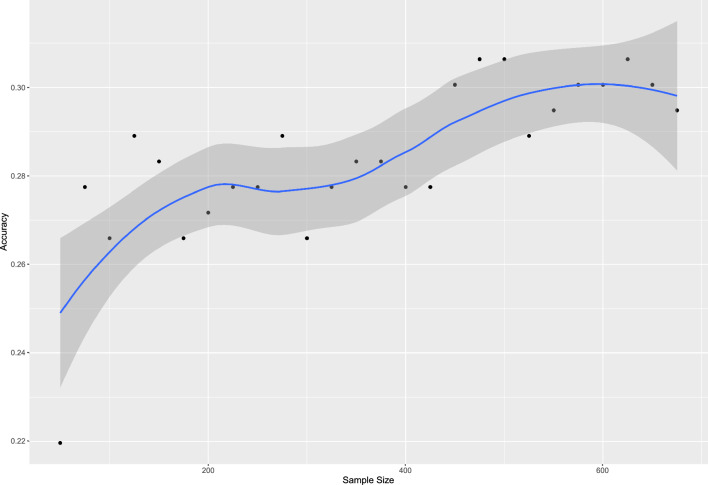
Simulation of increasing the training sample size in proportional odds logistic regression (POLR) models. Sample size increased accuracy of the POLR models up to a plateau at 400 before increasing again and plateauing at 600.

Simulation experiments with varying sample sizes demonstrated that increasing the sample size led to an increase in the accuracy of the proportional odds logistic regression (POLR) models. The accuracy continued to improve until it reached a plateau at 400 before increasing again, eventually plateauing at 600 (Fig. 7).

The POLR model has been made easily available in an online Shiny application.

## Discussion

In this study, we have examined the ability of different machine learning models in predicting all eight categories of the Glasgow Outcome Scale Extended (GOSE) by using IMPACT variables in a Swedish TBI cohort. We were also able to perform external validation in two different TBI cohorts, one from Leuven and the other from the ProTECT III study, which is a multicenter study performed in the USA. We found that a proportional odds logistic regression, random forest, and a neural network model perform with similar accuracy in the internal data. However, all models performed better in the external validation in the Leuven cohort but worse in the ProTECT III cohort. While this study lacks immediate clinical impact, it highlights the potential of predicting the full GOSE scale and provides a starting point for further studies. It is also, to our best knowledge, the first study to predict the full GOSE scale and utilize external validation in international cohorts.

TBI is a burdensome disease with oftentimes severe and lifelong implications for the patient, the family, and for society. Better prognostication is vital to further characterize the disease and to provide better care for these patients^19^. TBI outcome is usually assessed according to an 8-point scale in the Glasgow outcome scale-extended (GOSE)—1 (death) to 8 (upper good recovery)—however, it is usually dichotomized in dead/alive or favorable/unfavorable outcome. While outcome prediction models using machine learning after TBI have been extensively studied, previous studies have mainly focused on prediction of dichotomized outcome or mortality. A plethora of admission variables, both clinical, radiological and laboratory, have been used to develop machine learning models with excellent results in predicting mortality^20–23^. Some studies have focused on the connection between ICP, heart rate and mortality^24^ and others on the importance of using acuity scoring tools when predicting mortality after TBI^25^. Raj et al. have developed an ICP-MAP-CPP algorithm using a logistic regression approach that dynamically predicts mortality, showing improved performance of the models when adding the first three days of ICU variables^26,27^.

An advanced prognostic model that predicts all eight categories of GOSE could assist in both evaluating results from clinical trials as well as in providing exclusion criteria for patients that are either deemed so severely injured that they are beyond rescue or rather on track towards a full recovery with current standard of care. In both cases one might need to exclude these patients from a clinical trial as they otherwise potentially obscure treatment effects. Furthermore, as TBI is a very heterogeneous disease there is substantial variability in patient outcomes which makes it difficult to detect minor improvements from drug treatment which then requires very large study populations. In fact, adjusting for baseline characteristics using prognostic models have been found to increase statistical power and reduce the required sample size by 25–30%^28^. Prediction of the full GOSE scale nevertheless remains scarce. Bhattacharyay et al. utilized an exhaustive approach in threshold-level dichotomous prediction of all GOSE levels, and found that multinominal logistic regression, POLR, and a NN using a SoftMax output layer, performed similar when trained on admission data^29^. They found that adding predictors from the first 24 h of intensive care improved performance except in discriminating GOSE > 6 and > 7, suggesting that prediction of higher GOSE levels is more problematic than prediction of the lower levels. Furthermore, most previous studies lack external validation, which always means a risk of overfitting and lack of generalizability.

We have attempted to set up the evaluation of our models in accordance with what has been previously recommended. These recommendations include well defined and justified variables and that the models should have clear evaluation of discrimination and validation. Importantly, the models should also be externally validated on cohorts that differ in time or place to exclude over-fitting. The models should preferably be represented in a user-friendly fashion to be understandable and clinically available^3^.

Variables identified in the IMPACT study have been analyzed for their prognostic power and validated in many different cohorts of patients with moderate-severe TBI^19^. Age, injury severity and pathological findings on head CT-scans were found to have the highest R^2^ value. Furthermore, Maas et al. found high univariate odds ratios for age, low GCS-motor, pupil reactivity and CT-classification which is the main reason they were chosen for this study^19^. In our previous study we did not find that the laboratory variables of IMPACT (blood glucose and hemoglobin) increased the prognostic predictive power and thus excluded these from current study^30^.

### Evaluation of our models

The random forest and neural network models perform with higher accuracy in both internal and external data. All models also produce similar AUROC values in the range of 0.75–0.9 in the internal and Leuven data. This is similar to previous studies that performed external validation of the IMPACT model with AUROC values around 0.6–0.9^31–33^. The ProTECT III dataset posed greater challenges for the models developed within a single Swedish center, as evidenced by lower accuracy and AUROC values. This indicates the difficulty in predicting outcomes using these models on the ProTECT III data.

Comparing the accuracy in the Leuven cohort to the ProTECT III cohort we found accuracies in the range of 0.44–0.52 and 0.18–0.21 respectively. The models exhibited better predictive performances in foreseeing favorable outcomes for the Leuven patients but underestimated the outcomes of the ProTECT III patients. One explanation for this might be that the Leuven cohort had less patients with severe TBI compared to the ProTECT III cohort. The ProTECT III cohort also had a higher number of patients with abnormal pupillary reactions and generally worse GCS-motor scores. Although the Leuven cohort had significantly older patients compared to the training cohort and ProTECT had significantly younger patients, this had less impact on the predictions compared to the significantly lower GCS scores in the ProTECT cohort. Furthermore, the GCS scores in the ProTECT III studies were collected within four hours of the trauma, whereas the GCS scores in the Uppsala cohort were not controlled for the timepoint from trauma. Uppsala is a tertiary neurosurgical center with a large geographic uptake area and the mean time from trauma to GCS evaluation was 1.37 days (SD 12.8). Hypothesizing that the patients on average improved in GCS during the difference in time from trauma to examination between the Uppsala training data and ProTECT data, the patients with the same GOSE outcome would have lower GCS in the ProTECT cohort compared to the Uppsala cohort making the model more pessimistic in the ProTECT data. Furthermore, the ProTECT III data was collected from a clinical trial whereas the Uppsala and Leuven data were retrospectively collected from TBI registers. It is possible that the inclusion and exclusion criteria, as well as treatment regimens, contributed to the differences observed in the outcomes and the models predictive performances.

Upon further investigation, it was observed that the models, particularly the POLR model, exhibit a bias towards the most common categories in the training dataset. As a consequence, their predictions tend to be limited to GOSE categories 1, 3, and 8. This bias leads to high Total Category Proportion Discrepancy (TCPD) and Mean Category Proportion Discrepancy (MCPD) scores for the models. Interestingly, the more complex models, such as random forest (RF) and neural network (NN), exhibited finer-grained predictions. This suggests that these models may have captured and learned interactions within the data that the POLR model, with its simpler structure, could not capture effectively. The increased flexibility of RF and NN allows them to uncover and leverage intricate relationships in the data, leading to more nuanced predictions. Although the low accuracy and overprediction of some categories is suboptimal, it is perhaps understandable as other factors after admission should contribute to specifying patient outcome. It might be that accurate prediction of the full range of GOSE is not possible using only admission variables. The fact that there were class imbalances in GOSE categories in the training data also affected the models’ predictions. Balanced undersampling mitigates this issue by randomly removing instances from the majority classes to achieve a more balanced distribution. The information loss from the removed majority class cases could be mitigated by creating an ensemble of models which would include all data in at least some of the models. Creating an ensemble of POLR models improved prediction stratification at only slight cost of accuracy, as seen in Fig. 6.

Despite the calibration plots (Fig. 5) clearly indicating a lack of predicted probability in several GOSE categories for all models, the error distribution graphs suggest that the models are relatively balanced overall, especially in the internal and Leuven datasets. This observation is further supported by the relatively low MD and SSD.

In this setting, a simulation of the accuracy of POLR models with an increasing number in the sample size increased accuracy until it plateaued at around 600 patients. This could indicate that a larger number of patients might not increase accuracy of the POLR models further (Fig. 7). It is however probable that adding more variables to prediction models would require a higher number of patients, and cohorts with more evenly distributed GOSE outcome should be relevant in improving prediction of the middle categories. The choice of machine learning architecture and hyperparameters can also affect model performance. Here we explored several different versions, based on either classification trees or regression, and some that included ensemble learning. Most models performed roughly equal in terms of accuracy though, indicating that exploration of other machine learning models has limited potential to improve model performance. Including additional strong and independent predictors, on the other hand, has a clear potential to result in improved models. Previous studies have shown that the IMPACT variables only provide an R^2^ approaching 0.35 meaning that most contributing factors are not included^19^. In this study we chose to include the IMPACT admission variables as a starting point, but other variables could easily be added depending on the clinical setting wanted to be evaluated.

### Defining the optimal prediction model

TBI is a very heterogenous disease, and high accuracy in predicting the exactly correct category might be difficult using only admission data. Our models performed at best with a 35% accuracy in internal validation. Since high accuracy on the GOSE scale might be too challenging for the prediction models on these premises, it could be necessary to evaluate models trained on admission data using AW1 and AW2 instead.

The interpretability of machine learning models is important and higher interpretability facilitates understanding why and how the prediction has been made. Logistic regression generally has high interpretability, while neural networks and random forests generally have low interpretability. An outcome prediction model or calculator should also be user-friendly which will facilitate their clinical implementation. As proof of concept of improved availability, we chose to make the POLR model available as a Shiny application as it is a simple model suitable for the application. In the future, other more advanced models could be included as Shiny applications.

To summarize, the optimal model depends on the purpose of the model as well as the available data. For the evaluation of the care of TBI patients and the improvement of clinical studies, the model should have high accuracy and be well-calibrated. For practical reasons, the independent variables should also be routinely measured in the clinic to limit the need for data imputation to handle missing data.

### Strengths and limitations

One notable strength of our study is the external validation performed using both a cohort from Leuven and one from the multicenter ProTECT III study. However, the drawback in the fact that the models were developed within a single center remains. Although they demonstrated satisfactory performance during external validation in Leuven (single center), indicating potential generalizability, they exhibited poor performance in the ProTECT III data. Furthermore, while the models produce fairly balanced and accurate predictions on average, they all sometimes make predictions as far as seven categories from the observed outcomes which highlights the risk of applying the predictions in individual patient cases. Additionally, both the model training and validation were carried out using cohorts from high-income settings, and there is need for further investigations, particularly in developing countries. Utilizing the IMPACT variables offers several advantages. Firstly, the model built using these variables has undergone extensive validation, ensuring the reliability of these variables. Secondly, the variables are readily accessible, simplifying the data acquisition process which enabled us to avoid any data imputation procedures. This, however, means that the models presented here are unable to handle any missing data. In future settings, data selection and imputation might be necessary for complex models to be practical and usable. Another limitation was the relatively low rate of respondents for the outcome questionnaire in the Leuven database. It is reasonable to think that the non-responders could have worse clinical status, which could have led to selection bias in patient inclusion in this cohort.

## Conclusions

This is the first study predicting full scale GOSE outcome after TBI that includes external validation of the models in international cohorts. Furthermore, the POLR model have been made easily available as an online application. Our findings demonstrate the feasibility of developing and evaluating multicategory prediction models. However, achieving high accuracy in predicting all eight categories of the Glasgow Outcome Scale-Extended (GOSE) solely based on admission variables may not be possible. We also found that employing a confusion matrix is highly beneficial for presenting a concise visual representation of the overall performance of a multicategory prediction model. Additionally, considering accuracy within 1 and 2 categories as complementary measures can provide easily interpretable values that encompass predictions close to the observed outcome. For future studies on traumatic brain injury (TBI) outcome prediction and evaluating specific TBI interventions, neural networks, random forests, and ordinal regression models hold promise as potential approaches to explore. These models can help enhance the accuracy and effectiveness of TBI outcome prediction. It should be noted that great care should be taken when applying prognostic models that were developed from large patient cohorts into the clinical settings and when making decisions in the care of individual patients. Nevertheless, the fact is that clinicians make prognostic predictions using their clinical experience in everyday care. Tools that standardize these predictions would therefore be desirable, even though much work remains to realize this aspiration.

## Methods

### Data collection—model building cohort

All methods were carried out in accordance with relevant guidelines and regulations and were approved by the local committee as provided below.

The data collected at Uppsala University Hospital was approved by the Swedish Ethical review authority (Dnr 2010/138, 2020-05462, 2022-05456-01). Consent was obtained from all subjects included in the study. The models were developed using Swedish data collected in accordance with the ethics permit provided by the Uppsala university ethics committee (EPNU 2010/138) as well as the Swedish central ethics committee (CEPN 2010/138/1 and 2020–05462). For model training, 1157 patients with TBI admitted to the neurointensive care unit (NICU) at the Department of Neurosurgery at the University Hospital in Uppsala, Sweden, from 2008 to 2020, were screened for inclusion. Inclusion criteria were all TBI patients over 18 years of age that were admitted to the neurointensive care unit, regardless of trauma severity or pre-injury status. The only exclusion criteria were incomplete data. Clinical variables were extracted from the Uppsala Traumatic Brain Injury register^8^. The IMPACT core and radiological variables upon admission were used for modelling: age, pupil reaction, GCS-motor score and computed tomography (CT) classification^4^. Glasgow Outcome Scale (GOSE) was assessed at 6–12 months post-injury, by specially trained personnel in structured telephone interviews. The majority of patients (70%) was assessed between 6 and 8 months and 30% between 9 and 12 months. The Uppsala cohort was divided into a training set (80%) and a test set (20%). In order to have a balanced distribution of the dependent variable (GOSE) between training set and test set, the assignment was randomized with the constraint to fulfill this criterion.

The models were fine tuned in the training set using tenfold cross validation, before being evaluated using the test sets.

External validation was performed with data from Leuven, Belgium, and the US multicenter study Progesterone for Traumatic Brain Injury: Experimental Clinical Treatment (ProTECT III).

### Leuven TBI cohort

Since 2013, core data related to TBI (including admission GCS, pupil reaction, age, accident mechanism and admission CT Marshall score) for all patients of all TBI severities admitted to the University Hospitals Leuven, with or without hospitalization, are prospectively registered and kept in a secure database. Inclusion criteria were thus all consecutive TBI patients from 2013 until August 2022. Similar to the Uppsala cohort, no exclusion criteria were set for this cohort except incomplete data. At 6 months, patients received the GOSE postal questionnaire at home and the questionnaire was scored as previously published by Wilson et al.^9^. Response rate is regularly checked and was found to be around 38%. This project, intended for monitoring and benchmarking of quality of care and implemented in care standards, was approved by the UZ Leuven Ethics Committee. Data provided for the present study was fully anonymized. The Leuven cohort had a large majority of less severe TBI. The Leuven cohort had a mean admission GCS of 12.7 (SD 4.0) which was significantly higher than the training cohort (p < 0.001). The cohort had 380 (71%) mild TBI cases, 63 moderate (12%) and 92 (17%) severe TBI cases. Moreover, 76% of the patients presented with GCS-m 6 and 46% with the outcome of GOSE 8.

### ProTECT III study

ProTECT III was a phase 3 double blind placebo controlled multicenter clinical trial designed to determine the efficacy of administering intravenous (IV) progesterone (initiated within 4 h of injury and administered for 72 h, followed by an additional 24-h taper) versus placebo for treating patients with moderate to severe acute TBI (Glasgow coma scale score 12-4). The study was conducted in 49 high volume trauma centers participating in the National Institute of Neurological Disorders and Stroke (NINDS) and was conducted through the NINDS-funded Neurological Emergencies Treatment Trials (NETT) network in the United States, with tight adherence to guidelines on the management of moderate to severe TBI. Emory University Institutional Review Board was the IRB of record for the study with the protocol # IRB00014409. The term used in the US is—EFIC (Exception from Informed Consent) with is strictly guided by the FDA under the waiver of informed consent (21 CFR 50.24). It required community notification and consultation in all the areas where the study was being conducted, including local IRB review and approval at all sites. ProTECT III EFIC Plan submitted with FDA IND #104188.

The outcome of the trial was powered for a 10% increase in the proportion of patients with a favorable outcome by a 10% (absolute) difference, determined by the Glasgow Outcome Scale-Extended (GOSE) score at 6 months post injury when compared to placebo. The primary outcome analysis of the GOSE was a stratified dichotomy methodology for assessing improvement with GOSE scores as “favorable” versus “not favorable”, based on the brain injury severity score measured at randomization (best pre- randomization GCS or iGCS). 840 out of the planned 1,140 patients were randomized, as the study was stopped for futility at a preplanned analysis. The study was conducted under an exception from consent waiver, approved by the local IRBs and the FDA (Investigational New Drug application 104188). The ProTECT III cohort had more severely injured patients, with a mean admission GCS of 7.0 (SD 2.6) which was significantly lower than the training cohort (p < 0.001). No patients in the ProTECT cohort had mild TBI, 254 (30%) had moderate and 596 (70%) had severe TBI. Only 3% presented with GCS-m of 6 and 10% with had outcome of GOSE 8. Patient inclusion and exclusion in this cohort were much stricter than the Uppsala and Leuven cohorts. Specifically, inclusion criteria were moderate to severe injury (GCS 3–12 or motor response 2–5 if intubated), age ≥ 18, blunt and closed injury, as well as the ability to start drug infusion within 4 h from time of injury. Exclusion criteria were non-survivable trauma as determined by treating team, bilateral dilated unresponsive pupils, spinal cord injury with neurological deficits, pre-injury paralysis, inability to perform activities of daily living without assistance, cardiopulmonary arrest, status epilepticus on arrival or concern for post ictal state, systolic blood pressure < 90 at least 5 min apart, O_2_ saturation < 90 for at least 5 consecutive minutes, prisoner or ward of state, known active breast or reproductive organ cancers, known allergy to progesterone or Intralipid components, known history of blood clotting disorder or history of pulmonary embolism, blood or serum ethanol ≥ 250 mg %, positive qualitative pregnancy test, concern for inability to follow up at 6 months (residence in foreign country, homeless, undocumented immigrant, high likelihood of becoming incarcerated during study period etc.), and patient opt out. Excluded in this study were also patients with incomplete data.

All statistical modeling was done using R (version 4.1.2). The age and GCS scores of the cohorts were analyzed visually by histogram as well as by Shapiro-Wilks test and determined not to be normally distributed. Statistical comparison between the groups were thus conducted by the Wilcoxon rank-sum test.

### Proportional odds logistic regression

Logistic regression is used to model binary outcomes but can be extended to multiple category ordinal logistic regression when the response variable is measured on an ordinal scale. Furthermore, non-proportional odds logistic regression and proportional odds logistic regression can be utilized depending on whether the odds of moving to lower or higher categories is constant across all categories, the so-called parallel regression assumption. In order to determine whether to apply proportional odds logistic regression or non-proportional odds logistic regression we conducted a Brant test to verify that the parallel regression assumption holds. Subsequently, proportional odds logistic regression (POLR) was deemed suitable for modelling our data. As we utilized the well-validated IMPACT variables, no regularization was performed. The model was set up using the MASS package in R^10^.

### Random forest regression

Decision trees provide high accuracy in the data used to create them but are generally prone to overfitting. Random Forests (RFs) use an ensemble of decision trees to combine the probability estimates or predictions of the response variable from each tree, resulting in an improved overall prediction^11^. The optimal value chosen for the number of predictors sampled for splitting at each node (mtry) was found by testing different values and choosing the one with the least error rate in cross-validation. The mtry value was subsequently set to 1. The optimal number of trees for classification was found by plotting the error rate and the number of trees and was subsequently set to 500. The minimum samples required to split an internal node and the minimal samples in the terminal node size were left to default, meaning 2 and 1 respectively. The splitting criterion for classification in the randomForest package in R is Gini impurity. The random forest model was set up using the randomForest package in R^12^.

### Neural network regression

For this model we used a neural network with the SoftMax activation function as the output layer. This produces a predicted probability between 0 and 1 for each of the eight GOSE categories. The categorical data was transformed into one hot encoded data, and the numerical data (age) was normalized. The model was then set up with the Keras sequential model, including four hidden layers of 1600, 800, 200, and 100 nodes respectively with Rectified Linear Unit (ReLU) activation function and an output layer using the SoftMax activation function. Similar to the previous models, no regularization was performed. Batch size and number of epochs were set to 64 and 24, respectively. The loss function was set to categorical cross-entropy and the optimizer function was RMSprop. The neural network was set up using the Keras package in R^13^.

### Model evaluation

The performance of the models was determined by confusion matrices, error rates, calibration, and different aspects of prediction balance.

### Confusion matrices

A confusion matrix is a table that is often used to evaluate the performance of a classification model on a set of data for which the true values are known. It is particularly useful when predicting multiple outcome categories. The most classical example is the set with four entries: true positive, true negative, false positive and false negatives. Confusion matrices are commonly used when evaluating prediction models, for example the emotion recognition models by Carbonell et al^14^. In our context the matrix has eight rows and eight columns, for a total of 64 entries. Each entry represents the number of times the model predicted a given category, e.g. GOSE 1–8. It is then possible to create a heat map that represents the performance of the model in each category. Confusion matrices were created using the ggplot2^15^ and reshape^16^ packages in R.

### Error rates

When evaluating classification models, accuracy is a commonly used metric. It indicates the percentage of correct predictions made by the model in terms of categorizing the data. However, when predicting outcomes across eight categories, relying solely on accuracy might not suffice. Accuracy does not provide information about the extent of deviation from the correct category in incorrect predictions. To complement accuracy, we considered how far the models predictions deviate. In addition to overall accuracy, we also evaluated the model’s accuracy within one (AW1) category and accuracy within two categories (AW2) to gain a more nuanced understanding of its performance.

In order to achieve a comprehensive evaluation of our multiclass classification models, we averaged the values of average balanced accuracy, precision, recall, and specificity in the prediction of all GOSE categories.

Average balanced accuracy (ABA) reflects the model’s effectiveness in correctly classifying examples from multiple classes, while considering class imbalances in the data. ABA, the mean of sensitivity and specificity, ranges from 0 and 1, with higher scores indicating superior overall classification performance.

Precision evaluates the proportion of true positive predictions among all positive predictions. It was computed by dividing the number of true positives by the sum of true positives and false positives. Precision is particularly useful when the consequences of a false positive prediction are significant.

Recall (sensitivity) measures the proportion of true positives out of all actual positive examples in the dataset. It was computed by dividing the number of true positives by the sum of true positives and false negatives. Recall is most critical when the impact of missing a positive prediction (false negative) is substantial.

Finally, specificity determines the proportion of accurately predicted negatives. It was obtained by dividing the number of true negatives by the sum of true negatives and false positives. Thus, serving as an indicator of the accuracy of negative predictions.

### Area under the receiver operator curve

Although not suitable for evaluation of 8-level outcome prediction, the area under the receiver operator curve (AUROC) was used to assess the performance of the models. It was used to compare the classic dichotomized prediction of mortality (GOSE 1) versus survival (GOSE 2–8) and unfavorable outcome (GOSE 1–4) versus favorable outcome (GOSE 5–8) to evaluate the models' performance using test data. AUROC for mortality and unfavorable outcome were calculated using the pROC package in R^17^.

### Model calibration and balance

Model calibration represents how well the model’s predicted probabilities for different outcomes reflect the actual probability of those categories in the observed outcome. Calibration was displayed in calibration plots where the predicted probability was plotted against observed probability of that GOSE category. Calibration plots were calculated using the gbm package in R^13^.

To further evaluate model balance, several measures were defined and calculated.

Mean discrepancy (MD) is the average difference between the predictions and the observed outcomes. Negative values indicate that the model is too pessimistic (underestimation) and positive values that it is too optimistic (overestimation), while values close to zero indicate that the model is balanced in this regard.

Standard deviation discrepancy (SSD) measures the difference in variability between the predictions and the observed outcomes. Negative SSD values indicate that the predictions are less varied than the observations. Again, values close to zero indicate that the model is balanced in terms of variability.

Predicted Category Proportions (PCP) describes the fraction of patients that are predicted to be in each GOSE level while the Observed Category Proportions (OCP) describes the fraction that is actually observed in each level. Both PCP and OCP will therefore consist of 8 values for each model.

The PCP and OCP can be summarized by taking the sum of the absolute difference between the predicted category proportion and the observed category proportion for all GOSE levels, here referred to as the Total Category Proportion Discrepancy (TCPD). A TCPD value close to 0 indicates that the model, overall, neither favors nor disfavors certain categories while a value close to 2 means that the model tends to either overpredict or underpredict certain categories.

The Maximum Category Proportion Discrepancy (MCPD) represents the largest discrepancy among all GOSE levels. A value close to 0 indicates that the model neither favors nor neglects any of the categories, while a value close to 1 indicates that the displays significant bias towards or against a specific category.

Graphically, the balance of the models is displayed with category proportion graphs and error distribution graphs. Category proportion graphs display the proportion of observed outcomes compared to predicted outcomes, complementing the confusion matrices. Error distribution graphs display each predicted GOSE category value subtracted to the corresponding observed GOSE value. A value of 0 means balanced prediction, more negative values means that the model overestimated prognosis and positive values that the model underestimated prognosis.

### Balanced undersampling

In order to address the category imbalance in the data the models were trained using subsets of data generated by undersampling from the more common categories with the purpose of yielding a smaller, but balanced, training set. In other words, the more common categories were split between the models and the more uncommon were used in several models so that each model was trained on data with more equal spread between the GOSE categories. This means that less data is available for each model which is counteracted by training an ensemble of 10,000 POLR models on different subsets of the data. Analysis of the predicted probabilities by the models revealed a skewness in the probability distribution, and thus the final predicted probabilities were selected by taking the median of the predictions of all models.

### Sample size evaluation

With the purpose of determining whether a larger cohort of patients would affect the performance of our models, POLR models were set up using an increasing sample size to determine the relationship between the number of patients and accuracy.

### Model availability

The POLR model was made available using the Shiny package in R, where the IMPACT variables can be entered to predict GOSE outcome. The code is openly available on GitHub.

### Supplementary Information

Supplementary Table 1.

## Data Availability

The data that support the findings of this study are available from the corresponding author upon reasonable request. The code used for model setup is openly available on GitHub, https://github.com/DavidUAS/Rostamilab.git.

## References

[1] Dewan MC (2018). Estimating the global incidence of traumatic brain injury. J. Neurosurg..

[2] Perel P (2007). Prognosis following head injury: A survey of doctors from developing and developed countries. J. Eval. Clin. Pract..

[3] Perel P, Edwards P, Wentz R, Roberts I (2006). Systematic review of prognostic models in traumatic brain injury. BMC Med. Inform. Decis. Mak..

[4] Steyerberg EW (2008). Predicting outcome after traumatic brain injury: Development and international validation of prognostic scores based on admission characteristics. PLoS Med..

[5] Collaborators MCT (2008). Predicting outcome after traumatic brain injury: Practical prognostic models based on large cohort of international patients. BMJ.

[6] Eloranta S, Boman M (2022). Predictive models for clinical decision making: Deep dives in practical machine learning. J. Intern. Med..

[7] Muehlematter UJ, Daniore P, Vokinger KN (2021). Approval of artificial intelligence and machine learning-based medical devices in the USA and Europe (2015–20): A comparative analysis. Lancet Digit. Health.

[8] Nyholm L, Howells T, Enblad P, Lewen A (2013). Introduction of the Uppsala Traumatic Brain Injury register for regular surveillance of patient characteristics and neurointensive care management including secondary insult quantification and clinical outcome. Ups. J. Med. Sci..

[9] Wilson L (2021). A manual for the Glasgow Outcome Scale-extended interview. J. Neurotrauma.

[10] Venables WN, Ripley BD, Venables WN (2002). Modern Applied Statistics with S.

[11] James G, Witten D, Hastie T, Tibshirani R (2013). An Introduction to Statistical Learning : With Applications in R.

[12] Liaw AW (2002). M. Classification and regression by randomForest. R News.

[13] https://cran.r-project.org/web/packages/gbm/gbm.pdf.

[14] Fernandez Carbonell M, Boman M, Laukka P (2021). Comparing supervised and unsupervised approaches to multimodal emotion recognition. PeerJ Comput. Sci/.

[15] Wickham H (2016). ggplot2: Elegant Graphics of Data Analysis.

[16] Wickham H (2007). Reshaping data with the reshape package. J. Stat. Softw..

[17] Robin X (2011). pROC: An open-source package for R and S+ to analyze and compare ROC curves. BMC Bioinform..

[18] Wright DW (2014). Very early administration of progesterone for acute traumatic brain injury. N. Engl. J. Med..

[19] Lingsma HF, Roozenbeek B, Steyerberg EW, Murray GD, Maas AI (2010). Early prognosis in traumatic brain injury: from prophecies to predictions. Lancet Neurol..

[20] Abujaber A (2020). Prediction of in-hospital mortality in patients with post traumatic brain injury using National Trauma Registry and Machine Learning Approach. Scand. J. Trauma Resusc. Emerg. Med..

[21] Matsuo K (2020). Machine learning to predict in-hospital morbidity and mortality after traumatic brain injury. J. Neurotrauma.

[22] Lang L (2023). An independently validated nomogram for individualised estimation of short-term mortality risk among patients with severe traumatic brain injury: A modelling analysis of the CENTER-TBI China Registry Study. EClinicalMedicine.

[23] Amorim RL (2019). Prediction of early TBI mortality using a machine learning approach in a LMIC population. Front. Neurol..

[24] Dimitri GM (2022). Modeling brain-heart crosstalk information in patients with traumatic brain injury. Neurocrit. Care.

[25] Najafi Z, Zakeri H, Mirhaghi A (2018). The accuracy of acuity scoring tools to predict 24-h mortality in traumatic brain injury patients: A guide to triage criteria. Int. Emerg. Nurs..

[26] Raj R (2019). Machine learning-based dynamic mortality prediction after traumatic brain injury. Sci. Rep..

[27] Raj R (2022). Dynamic prediction of mortality after traumatic brain injury using a machine learning algorithm. NPJ Digit. Med..

[28] Hernandez AV (2005). Subgroup analysis and covariate adjustment in randomized clinical trials of traumatic brain injury: A systematic review. Neurosurgery.

[29] Bhattacharyay S (2022). The leap to ordinal: Detailed functional prognosis after traumatic brain injury with a flexible modelling approach. PLoS One.

[30] Rostami E (2022). Prognosis in moderate-severe traumatic brain injury in a Swedish cohort and external validation of the IMPACT models. Acta Neurochir. (Wien).

[31] Roozenbeek B (2012). Predicting 14-day mortality after severe traumatic brain injury: application of the IMPACT models in the brain trauma foundation TBI-trac(R) New York State database. J. Neurotrauma.

[32] Roozenbeek B (2012). Prediction of outcome after moderate and severe traumatic brain injury: External validation of the International Mission on Prognosis and Analysis of Clinical Trials (IMPACT) and Corticoid Randomisation After Significant Head injury (CRASH) prognostic models. Crit. Care Med..

[33] Sun H, Lingsma HF, Steyerberg EW, Maas AI (2016). External validation of the international mission for prognosis and analysis of clinical trials in traumatic brain injury: Prognostic models for traumatic brain injury on the study of the neuroprotective activity of progesterone in severe traumatic brain injuries trial. J. Neurotrauma.

