# Supplementary material for: Refining outcome prediction after traumatic brain injury with machine learning algorithms
Source: Sci Rep. 2024 Apr 5;14:8036. doi: 10.1038/s41598-024-58527-4 (PMC10997790; doi:10.1038/s41598-024-58527-4)
Supplement: Supplementary file 1 — Supplementary Table 1. [file 41598_2024_58527_MOESM1_ESM.docx]

|  | **POLR** | **RF** | **NN** |
| --- | --- | --- | --- |
| Accuracy fold 1 | 0.29 | 0.32 | 0.28 |
| Accuracy fold 2 | 0.33 | 0.32 | 0.26 |
| Accuracy fold 3 | 0.34 | 0.29 | 0.24 |
| Accuracy fold 4 | 0.36 | 0.31 | 0.23 |
| Accuracy fold 5 | 0.37 | 0.39 | 0.25 |
| Accuracy fold 6 | 0.31 | 0.3 | 0.35 |
| Accuracy fold 7 | 0.31 | 0.33 | 0.27 |
| Accuracy fold 8 | 0.4 | 0.29 | 0.35 |
| Accuracy fold 9 | 0.24 | 0.35 | 0.33 |
| Accuracy fold 10 | 0.32 | 0.28 | 0.33 |
|  |  |  |  |
| Mean accuracy | 0.32 | 0.32 | 0.29 |
| Standard deviation | 0.04 | 0.03 | 0.05 |

Supplementary table 1. Accuracy of all ten folds from the cross-validation in the Uppsala training set. The mean accuracy were consistent between all models in the range of 0.29-0.32.
